# Anti-inflammatory and Antioxidant Activity of Ocimum tenuiflorum- and Stevia rebaudiana-Mediated Silver Nanoparticles: An In Vitro Study

**DOI:** 10.7759/cureus.50109

**Published:** 2023-12-07

**Authors:** Indumathy Pandiyan, Meignana Arumugham I, Srisakthi D, Rajeshkumar Shanmugam

**Affiliations:** 1 Department of Public Health Dentistry, Saveetha Dental College and Hospitals, Saveetha Institute of Medical and Technical Sciences, Saveetha University, Chennai, IND; 2 Nanobiomedicine Lab, Centre for Global Health Research, Saveetha Medical College and Hospitals, Saveetha Institute of Medical and Technical Sciences, Saveetha University, Chennai, IND; 3 Department of Pharmacology, Saveetha Dental College and Hospitals, Saveetha Institute of Medical and Technical Sciences, Saveetha University, Chennai, IND

**Keywords:** stevia rebaudiana, silver nanoparticles, ocimum tenuiflorum, healthy lives, health, anti-inflammatory, antioxidant

## Abstract

Background: Nanotechnology, a field bridging material science and biology, explores various applications. Silver nanoparticles, ranging from 1 nm to 100 nm, are commonly labeled as "silver," although some contain a substantial quantity of silver oxide owing to the heightened ratio of silver atoms on the surface compared to the bulk. This characteristic establishes silver as a prominent nanoparticulate material. *Stevia*, valued for its leaf's sweetness and purported therapeutic qualities, has been utilized for centuries in South America functioning both as a natural sweetener and in traditional health remedies. The objective of this study was to evaluate the anti-inflammatory and antioxidant activity of *Ocimum tenuiflorum*- and *Stevia rebaudiana*-mediated silver nanoparticles.

Methods: The methods employed involved evaluating the anti-inflammatory effects using the bovine serum albumin (BSA) assay and antioxidant effects using the 2,2-diphenyl-1-picrylhydrazyl (DPPH) assay, with varying concentrations (10 µL, 20 µL, 30 µL, 40 µL, and 50 µL) of the nanoparticles.

Results: The results indicated that the anti-inflammatory properties of the nanoparticles surpassed standard values at concentrations of 10 µL, 20 µL, and 30 µL, while the antioxidant properties were also notably surpassing standard values at equivalent concentrations. The maximum inhibition percentage was noted with 10 µL (72.5%).

Conclusion: The silver nanoparticles, fortified with extracts from *Ocimum tenuiflorum* and *Stevia rebaudiana*, exhibited a promising potential as effective anti-inflammatory and antioxidant agents, suggesting their viability as alternatives to commercially available products.

## Introduction

The heightened interest in medicinal plants is a result of the increasing scientific support for the health benefits obtained from extracts and phytochemicals found in botanical sources [1]. Numerous bioactive compounds within these plants have demonstrated therapeutic utility in addressing chronic disorders, including cancer, cardiovascular disease, and neurodegenerative conditions [2]. This therapeutic efficacy is often ascribed to their multifaceted biological activities, encompassing antioxidant, antibacterial, anticancer, antifungal, and antiviral characteristics; they exhibit the capacity to modulate cellular activities in inflammation-related cells like mast cells, macrophages, lymphocytes, and neutrophils [1,2]. The phenomenon of metal accumulation in certain plants, recognized as hyperaccumulators, can attain significant levels, rendering them suitable for metal extraction applications. For instance, a ton of milfoil's green mass has the potential to yield more than 1 kg of zinc [3].

Despite the long-standing awareness of the geobiology of plants and the metal deposition capabilities of microorganisms, the application of plant-derived extracts in the synthesis of metal nanoparticles has experienced accelerated adoption following heightened economic and environmental concerns associated with conventional physical and chemical methodologies. This shift marks the initiation of a new era [4]. The biosynthesis of nanoparticles presents several advantages over traditional physical and chemical approaches. In contrast to alternative methods that employ toxic chemicals deemed unsuitable for medical applications, biological synthesis methods leverage microorganisms such as bacteria, yeast, fungi, or plants, all of which are inherently present in natural recycling processes. Moreover, biological processes exhibit remarkably low overall material and energy consumption, thereby establishing them as a cost-effective and environmentally sustainable alternative [5]. Enzymes and proteins, either naturally occurring in the environment as byproducts of common microorganism activities or induced upon contact with specific metal ions, have been documented as key entities responsible for the intra- or extracellular production of nanoparticles in biological methodologies involving microorganisms [6].

Silver nanoparticles (AgNPs) encompass particles with dimensions ranging from 1 nm to 100 nm. Although commonly referred to as "silver," their classification is attributable to the substantial ratio of surface to bulk silver atoms, with certain instances featuring a notable presence of silver oxide. The fabrication of nanoparticles can yield diverse shapes tailored to specific applications. The attributes of AgNPs that hold relevance to human treatments are currently under scrutiny through laboratory and animal studies, with a focus on evaluating their potential efficacy, toxicity, and associated costs [7].

*Stevia* (*Stevia rebaudiana Bertoni*) has a historical legacy as a natural sweetener and an integral component of traditional medicine in South America, spanning several centuries, owing to the sweetness and purported therapeutic attributes inherent in its leaves [8]. The sweet taste of *Stevia* is ascribed to various glycosides, including stevioside, rebaudiosides A, B, C, D, and E, and dulcoside A [9]. These inherent sweeteners of natural origin demonstrate medicinal effectiveness against a variety of conditions, including diabetes mellitus, candidiasis, hypertension, inflammation, obesity, and cancer, among other ailments. Furthermore, *Stevia* contains a diverse array of metabolites, including flavonoids, alkaloids, water-soluble chlorophylls, xanthophylls, hydroxycinnamoyl derivatives (such as caffeoyl and chlorogenic acid derivatives), neutral water-soluble oligosaccharides, free sugars, amino acids, lipids, essential oils, and trace elements, which contribute to its therapeutic repertoire. Noteworthy among these are austroinullin, recognized for its vasodilator and cardiotonic properties, along with anaesthetic and anti-inflammatory agents [10].

However, the biosynthesis of AgNPs from *Ocimum tenuiflorum* and *Stevia rebaudiana*, both extensively employed in folk medicine since ancient times, remains undocumented. Consequently, the current study aims to pioneer the development of *Ocimum tenuiflorum*- and *Stevia rebaudiana*-mediated AgNPs. The primary objective is to systematically investigate and analyze the collective anti-inflammatory and antioxidant activities inherent in the biosynthesized AgNPs derived from *Ocimum tenuiflorum* and *Stevia rebaudiana.*

## Materials and methods

An in vitro study was conducted at Saveetha Dental College and Hospitals, Saveetha Institute of Medical and Technical Sciences, Chennai, India, to evaluate the anti-inflammatory and antioxidant property of *Ocimum tenuiflorum*- and *Stevia rebaudiana*-mediated AgNPs. Study approval was granted by the Scientific Review Board (SRB/SDC/FACULTY/23/PHD/141) and Institutional Ethics Committee (IEC/SDC/FACULTY/23/PHD/292) of Saveetha University, ​​ensuring adherence to ethical guidelines. 

Collection and preparation of plant

Fresh micronized leaves of *Ocimum tenuiflorum* and *Stevia rebaudiana* were procured from the South Indian market, meticulously determined and substantiated by botanists. Subsequently, the powdered specimens of *Ocimum tenuiflorum *and *Stevia rebaudiana* were individually stored in hermetically sealed containers. For the preparation of the aqueous extract, 500 mg of each powdered plant material was combined with 100 mL of distilled water and subjected to a boiling process for 20 minutes within the temperature range of 70-80°C, employing a heating mantle. Following the boiling step, the resulting extract underwent filtration utilizing Whatman No. 1 filter paper (Cytiva, Marlborough, Massachusetts, United States) and allowing it to stand undisturbed for 20 minutes.

Preparation of AgNP extract

**Figure 1:**
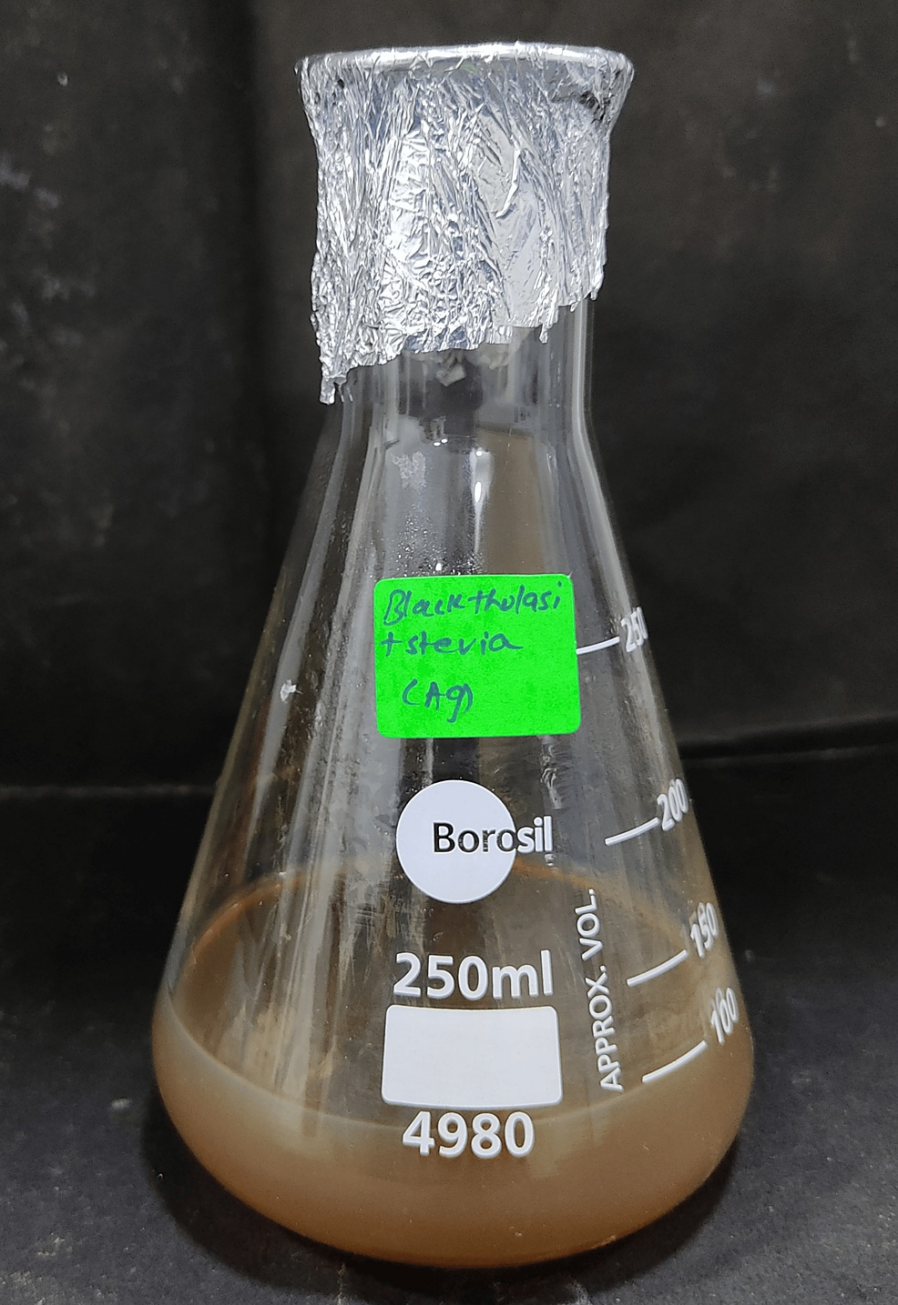
Mixture of silver nitrate solution with the plant extract

Commencing the production of the extract with AgNPs, 30 mM solution of silver nitrate was precisely measured and combined with 60 mL of distilled water. The resulting silver nitrate solution was then blended with 40 mL of the previously filtered plant extract, as illustrated in Figure 1. This amalgamation was allowed to stand on a magnetic stirrer for one hour, operating at a speed of 380-420 revolutions per minute (rpm), and concurrently placed utilizing a shaker container to facilitate particle intermixing for the purpose of green synthesis. The reduction of silver nitrate to AgNPs was systematically monitored using UV spectrometers at 24-hour intervals over a duration of three days. Visual observation of color changes was noted and documented photographically. Subsequently, the AgNP solution underwent centrifugation at 8000 rpm for 10 minutes using a Lark refrigerated centrifuge (Lark Innovation Fine Technology, Chennai, Tamil Nadu, India). The resulting pellet was collected, subjected to two consecutive washes with distilled water, and then purified. The final purified pellet was retrieved and subjected to drying subjected to temperatures between 100°C and 150°C for a period of 24 hours. Finally, the dried nanoparticle powder was collected and meticulously stored in an airtight Eppendorf tube (Eppendorf, Hamburg, Germany).

Determination of anti-inflammatory activity using the albumin denaturation assay

Test Group (Ocimum tenuiflorum- and Stevia rebaudiana-Mediated AgNPs)

**Figure 2:**
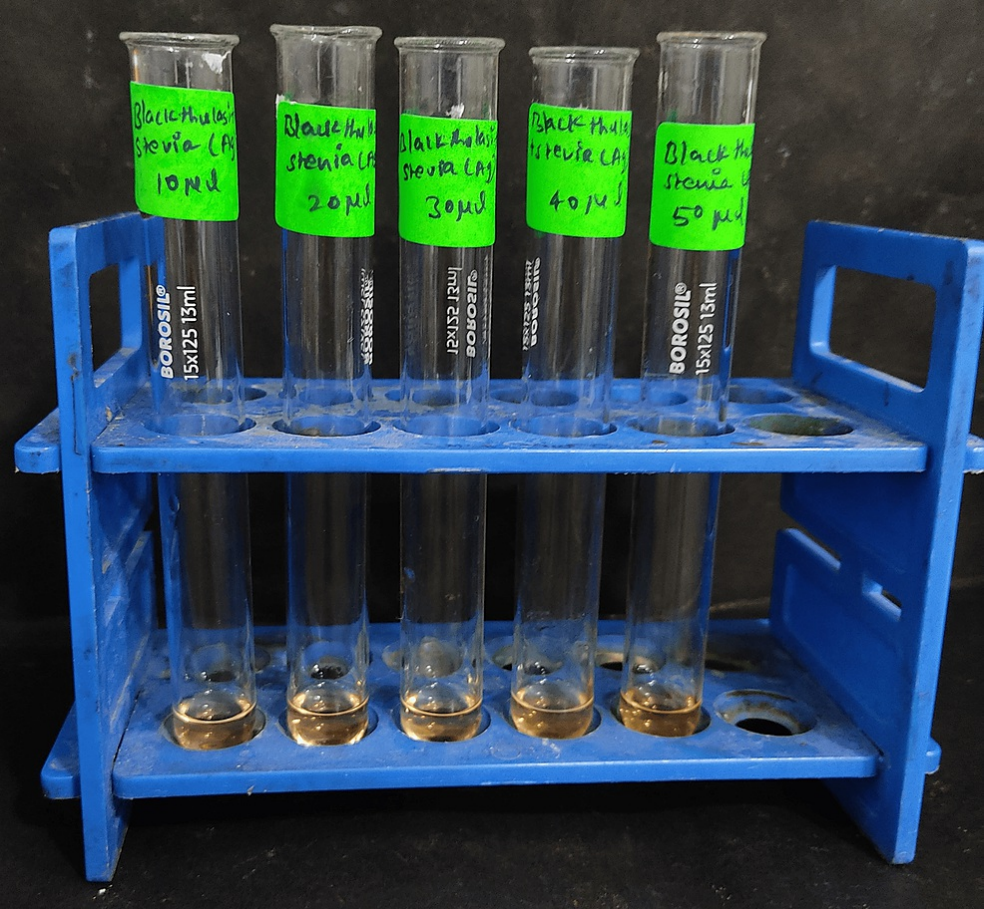
Anti-inflammatory activity using the albumin denaturation assay

In five separate test tubes labeled according to the volume of nanoparticles (10 µL, 20 µL, 30 µL, 40 µL, and 50 µL, as depicted in Figure 2), aliquots of the respective nanoparticle volumes were dispensed. To each of these test tubes, 2 mL of a 1% bovine serum albumin (BSA) solution was introduced. Subsequently, volumes of distilled water corresponding to 390 µL, 380 µL, 370 µL, 360 µL, and 350 µL were added to the test tubes containing 10 µL, 20 µL, 30 µL, 40 µL, and 50 µL of nanoparticles, respectively. For the control group, a solution comprising 2 mL of dimethyl sulfoxide was incorporated into 2 mL of the BSA solution.

Standard Group (Diclofenac Sodium)

For comparison analysis with the standard group, a discrete portion of diclofenac sodium, which is a nonsteroidal anti-inflammatory drug (NSAID) renowned for its potent anti-inflammatory attributes, was utilized. This pharmacological agent operates through the inhibition of cyclooxygenase (COX) enzymes, with a particular emphasis on COX-2. These enzymes play a pivotal role in the biosynthesis of pro-inflammatory prostaglandins. By blocking these enzymes, diclofenac reduces the production of inflammatory mediators leading to its anti-inflammatory effects. This known mechanism of action was used as a base for comparison making it a reliable reference point when evaluating the anti-inflammatory potential of other substances. Five separate test tubes were filled with diclofenac sodium in volumes of 10 µL, 20 µL, 30 µL, 40 µL, and 50 µL, respectively. Subsequently, 2 mL of a 1% BSA solution was introduced into each test tube. Incubation of the test tubes occurred at room temperature for a duration of 10 minutes, followed by an additional incubation period in a water bath set at 55°C for approximately 10 minutes. Subsequently, the absorbance levels were measured at 660 nm using a UV spectrophotometer.

Evaluation of antioxidant activity

Test Group (Ocimum tenuiflorum- and Stevia rebaudiana-Mediated AgNPs)

In five distinct test tubes, volumes of the nanoparticle solution were dispensed, specifically 10 µL, 20 µL, 30 µL, 40 µL, and 50 µL, respectively. To each of these test tubes, 1 mL of 2,2-diphenyl-1-picrylhydrazyl (DPPH) was introduced. Concurrently, volumes of 50% methanol solution corresponding to 1990 µL, 1980 µL, 1970 µL, 1960 µL, and 1950 µL were added to the test tubes containing 10 µL, 20 µL, 30 µL, 40 µL, and 50 µL of nanoparticles, respectively. For the control group, a solution comprising 1 mL of DPPH was incorporated into 2 mL of a methanol solution.

Standard Group (Ascorbic Acid)

Ascorbic acid was used as standard. The test tubes were incubated in a dark cupboard for around 20 minutes. Absorbance was measured at 517 nm in the UV spectrophotometer. Spectrophotometry is a quantitative method which enables the measurement of light absorption by a substance in a sample. It is known for its rapid and efficient analysis, allowing for swift measurements. Importantly, spectrophotometry is non-destructive, preserving the sample as it does not consume it during the measurement process.

The percentage inhibition was calculated using the formula (Control OD-(Sample OD/Control OD))×100 where Control OD is the absorbance of the negative control and Sample OD is the absorbance of the test sample.

**Figure 3:**
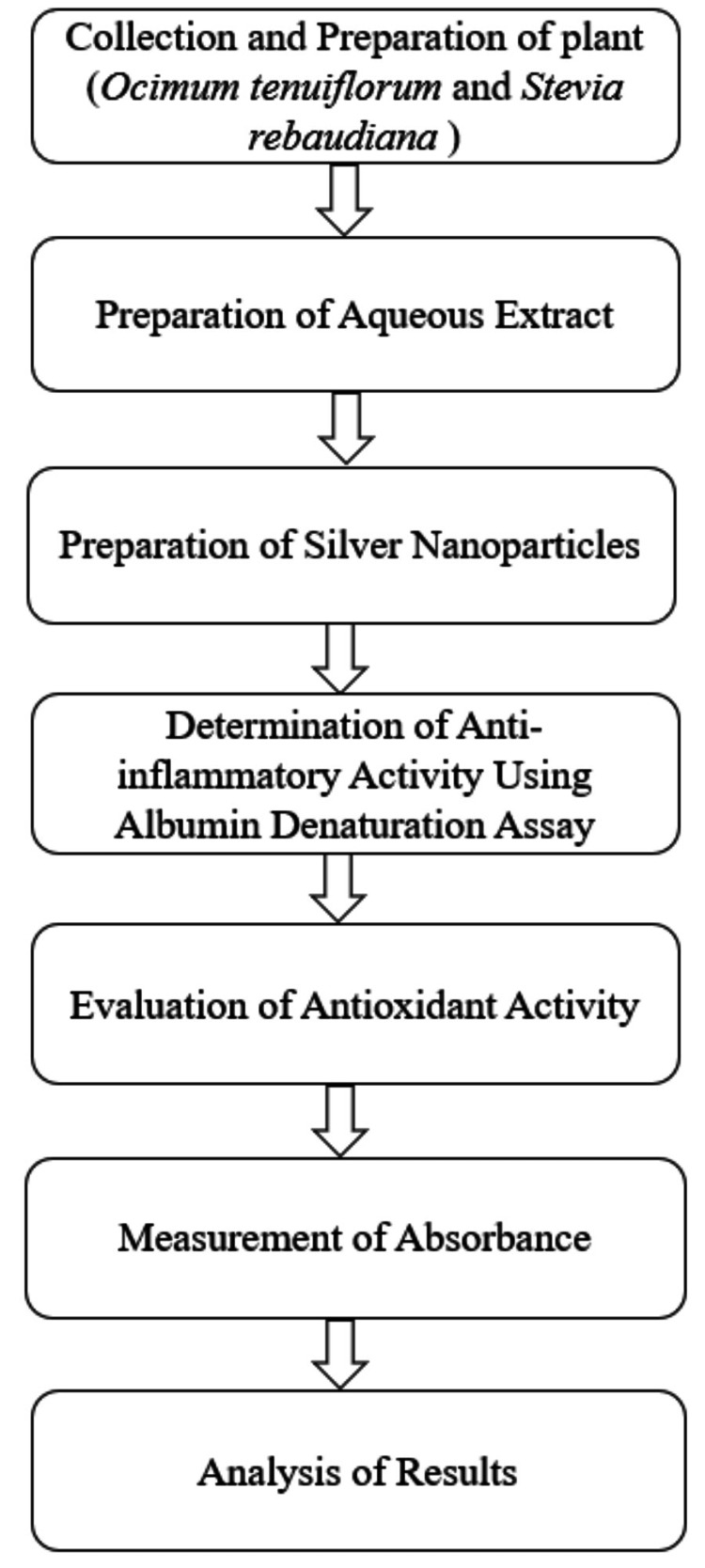
Flowchart of the methodology

The flowchart of the methodology is illustrated in Figure 3. This figure outlines the sequential steps involved in the determination of anti-inflammatory activity using the albumin denaturation assay, the evaluation of antioxidant activity, and the final measurement of absorbance at 517 nm.

## Results

**Figure 4:**
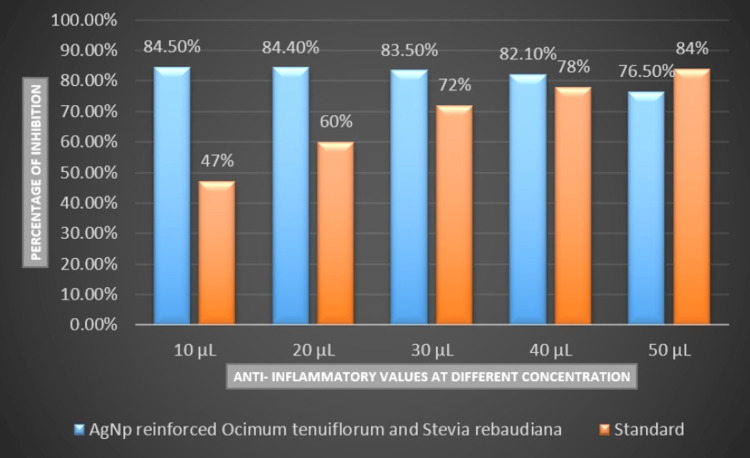
Anti-inflammatory attributes of silver nanoparticles incorporating Ocimum tenuiflorum and Stevia rebaudiana extract at various concentrations

The assessment of anti-inflammatory values indicated the heightened efficacy of the nanoparticles at all concentrations. The percentage of inhibition reached its peak at the 50 µL concentration (92.6%), followed by 84.5% at 10 µL and 84.4% at 20 µL. Figure 4 illustrates the anti-inflammatory attributes of AgNPs incorporating *Ocimum tenuiflorum* and *Stevia rebaudiana* extract at various concentrations, juxtaposed against standard values.

**Figure 5:**
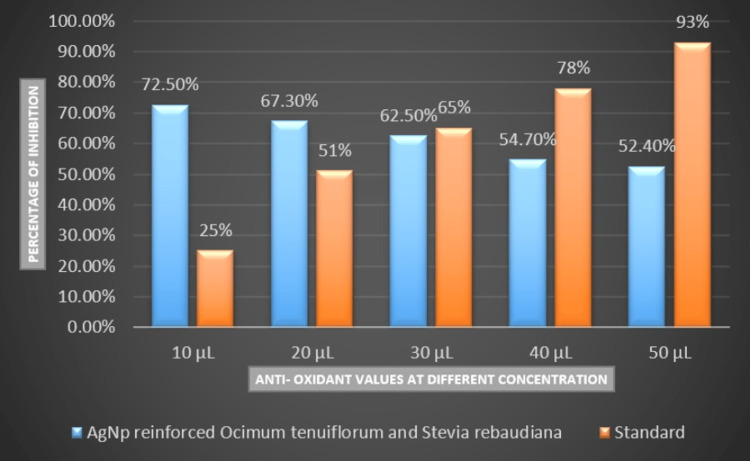
Antioxidant properties of silver nanoparticles fortified with extracts from Ocimum tenuiflorum and Stevia rebaudiana across various concentrations

Regarding antioxidant properties, the nanoparticles demonstrated superiority over standard values at concentrations of 10 µL and 20 µL. The percentage of inhibition was notably high at the 10 µL concentration (72.5%), followed by 67.3% at 20 µL. Subsequently, a decline in inhibition percentages was observed at 40 µL (54.7%) and 50 µL (52.4%). Figure 5 illustrates the antioxidant properties of AgNPs fortified with extracts from *Ocimum tenuiflorum* and *Stevia rebaudiana* across various concentrations in comparison to standard values.

## Discussion

Eugenol (1-hydroxy-2-methoxy-4-allylbenzene), identified as an active compound in *Ocimum sanctum*, is believed to be a key contributor to the therapeutic efficacy of tulsi [11]. Additionally, two other vital components, ursolic acid and carvacrol, play a significant role in imparting antibacterial properties to tulsi. The observed antimicrobial activity of tulsi is attributed to the presence of these constituents. In this study, we utilized leaves from both the common tulsi and the sweet tulsi varieties.

The primary phenolic components identified in both fresh and dried *Stevia* leaves include chlorogenic acid, trans-ferulic acid, rutin, and caffeic acid, with their abundance following the order mentioned. The occurrence of chlorogenic acid, caffeic acid, and rutin, with a notable prevalence of chlorogenic acid, aligns with previously reported findings on the composition of *Stevia* extracts [1,12]. These findings contribute to the understanding of the chemical composition of tulsi and *Stevia* leaves, shedding light on their potential therapeutic properties and health benefits.

Chlorogenic acid typically predominates among its isomers, and its prevalence in various foods makes it one of the polyphenols frequently found in human diets [13]. Polyphenols, including chlorogenic acid, constitute a crucial antioxidant group known for their pharmacological properties. These compounds showcase a spectrum of positive impacts, including anti-inflammatory, anticancer, anti-obesity, antiviral, antimicrobial, anti-lipidemic, anti-diabetic, anti-hypertensive, and anti-neurodegenerative properties [14]. The intricate mechanisms underlying these diverse activities are continually under investigation and are poised to significantly impact the pharmacological understanding of bioactive compounds derived from *Stevia* [15,16].

In this investigation, the AgNPs exhibited a distinct peak at 450 nm. Notably, these nanoparticles demonstrated potent anti-inflammatory effects at lower concentrations, while their antioxidant activity was more pronounced at higher concentrations. This could be because of the dose-dependent cellular responses. Many biological responses to nanoparticles exhibit dose-dependent effects. At lower concentrations, the nanoparticles may trigger specific anti-inflammatory pathways, leading to a potent anti-inflammatory response. At higher concentrations, the biological response may shift, and the nanoparticles could engage with different cellular pathways, including those related to antioxidant defense mechanisms. Also, nanoparticles may exhibit different levels of biocompatibility and toxicity at varying concentrations. At lower concentrations, the nanoparticles may be well-tolerated, promoting anti-inflammatory effects [17,18]. Higher concentrations might surpass a threshold where toxicity becomes more pronounced, inducing cellular stress and activating antioxidant defense mechanisms as a response to the increased oxidative burden.

Tulsi, with its diverse therapeutic applications, showcases significant clinical effects spanning various domains, many of which are linked by inflammation. Several in vitro and in vivo studies have reported the anti-inflammatory properties of tulsi, suggesting the presence of bioactive secondary metabolites that act independently or synergistically to mitigate inflammatory pathways. Furthermore, tulsi may serve as a valuable supplement to pharmacotherapy and nutrition in the context of treating metabolic diseases, potentially reducing the reliance on high doses of medications that may carry undesirable side effects [19,20].

However, it is crucial to acknowledge the limitations of this study, particularly its in vitro nature. Consequently, caution is warranted in extrapolating the results of anti-inflammatory and antioxidant activities to their potential clinical effectiveness. Further research, including clinical studies, is essential to bridge this gap and provide a more comprehensive understanding of the therapeutic implications of these findings.

Recommendations

Subsequent investigations should advance to in vivo trials involving animal models, providing a critical bridge between in vitro findings and potential human applications. Moving forward, it is advisable to progress to clinical trials, taking into consideration human acceptance values and safety considerations. This sequential approach, starting with animal studies and then transitioning to human trials, will contribute to a more comprehensive understanding of the practical implications and safety profile of the AgNPs and their associated bioactive compounds. Conducting clinical trials with due consideration to ethical and regulatory standards will be pivotal in evaluating the translational potential of the observed anti-inflammatory and antioxidant effects in a real-world context.

## Conclusions

The outcomes of this study indicate that AgNPs strengthened with *Ocimum tenuiflorum* and *Stevia rebaudiana* extract exhibit significant potential as both anti-inflammatory and antioxidant agents. These findings suggest a promising alternative to commercially available products in the realm of therapeutic interventions. The observed properties of these nanoparticles, coupled with their natural origin, pave the way for the further exploration of their applications in medical and healthcare contexts, with potential implications for the development of novel and effective interventions.
